# Physical and Chemical Properties of Vegetable Films Based on Pumpkin Purée and Biopolymers of Plant and Animal Origin

**DOI:** 10.3390/molecules28124626

**Published:** 2023-06-07

**Authors:** Monika Janowicz, Justyna Kadzińska, Joanna Bryś, Agnieszka Ciurzyńska, Magdalena Karwacka, Sabina Galus

**Affiliations:** 1 Department of Food Engineering and Process Management, Institute of Food Sciences, Warsaw University of Life Sciences—SGGW, 159c Nowoursynowska St., 02-776 Warsaw, Poland; jk.kadzinska@gmail.com (J.K.); magdalena_karwacka@sggw.edu.pl (M.K.); 2 Division of Organic and Food Chemistry, Department of Chemistry, Institute of Food Sciences, Warsaw University of Life Sciences—SGGW, 159c Nowoursynowska St., 02-776 Warsaw, Poland; joanna_brys@sggw.edu.pl

**Keywords:** edible films, pumpkin, gelatin, pectin, edible packaging, thermal properties, sorption, tensile strength

## Abstract

Highly methylated apple pectin (HMAP) and pork gelatin (PGEL) have been proposed as gelling agents for pumpkin purée-based films. Therefore, this research aimed to develop and evaluate the physiochemical properties of composite vegetable films. Granulometric analysis of film-forming solutions showed a bimodal particle size distribution, with two peaks near 25 µm and close to 100 µm in the volume distribution. The diameter *D*_4.3_, which is very sensitive to the presence of large particles, was only about 80 µm. Taking into account the possibility of creating a polymer matrix from pumpkin purée, its chemical characteristic was determined. The content of water-soluble pectin was about 0.2 g/100 g fresh mass, starch at the level of 5.5 g/100 g fresh mass, and protein at the level of about 1.4 g/100 g fresh mass. Glucose, fructose, and sucrose, the content of which ranged from about 1 to 1.4 g/100 g fresh mass, were responsible for the plasticizing effect of the purée. All of the tested composite films, based on selected hydrocolloids with the addition of pumpkin purée, were characterized by good mechanical strength, and the obtained parameters ranged from about 7 to over 10 MPa. Differential scanning calorimetry (DSC) analysis determined that the gelatin melting point ranged from over 57 to about 67 °C, depending on the hydrocolloid concentration. The modulated differential scanning calorimetry (MDSC) analysis results exhibited remarkably low glass transition temperature (Tg) values, ranging from −34.6 to −46.5 °C. These materials are not in a glassy state at room temperature (~25 °C). It was shown that the character of the pure components affected the phenomenon of water diffusion in the tested films, depending on the humidity of the surrounding environment. Gelatin-based films were more sensitive to water vapor than pectin ones, resulting in higher water uptake over time. The nature of the changes in water content as a function of its activity indicates that composite gelatin films, with the addition of pumpkin purée, are characterized by a greater ability to adsorb moisture from the surrounding environment compared to pectin films. In addition, it was observed that the nature of the changes in water vapor adsorption in the case of protein films is different in the first hours of adsorption than in the case of pectin films, and changes significantly after 10 h of the film staying in an environment with relative humidity RH = 75.3%. The obtained results showed that pumpkin purée is a valuable plant material, which can form continuous films with the addition of gelling agents; however, practical application as edible sheets or wraps for food products needs to be preceded with additional research on its stability and interactions between films and food ingredients.

## 1. Introduction

The food industry is currently showing interest in edible packaging technology with films and coatings, which results from the growing popularity of convenient and minimally processed food, the need for extending shelf life with mild technology, and more sustainable types of food packaging [1,2,3]. The complete replacement of synthetic polymers is not possible with the current state of knowledge and technology, not only due to the too-low barrier of edible films and coatings against water vapor and their relatively poor mechanical properties, but also due to the high risk of microbial contamination during food production and distribution [4,5,6]. Intensive interdisciplinary scientific and technological research is being carried out, aimed at introducing edible films as independent structures to the market on a large scale. A good example of which is the already existing on-the-market protein and starch casings for sausages, which are an alternative to conventional casings [3,7,8,9].

The properties of edible films differ depending on the type of polymers used. The basic factor limiting the wide application of this type of structure is their mechanical strength and hygroscopicity. Therefore, intensive research is carried out to improve the properties of edible packaging and the possibility of fulfilling additional functions, e.g., functional, utilitarian, or nutritional [10]. The enhancement of edible films has become a pressing matter in recent times. To achieve this, one must combine various components [7,11] with film-forming properties, create composites, incorporate essential oils into the polymer matrix [12,13], and leverage the latest nanotechnology advancements to produce edible packaging [14]. However, an equally promising trend in the production of edible films seems to be the possibility of introducing fruit and vegetable purées into the polymer matrix, which are a rich source of ingredients with film-forming properties, such as proteins, pectins, starch, cellulose, and its derivatives [15]. In addition, the gelification process of hydrocolloids leads to a more stable dispersion and needs to be observed [16]. The research performed for starch/pectin-based film-forming dispersions showed that after the initial coalescence, the stabilization of each dispersion depends on polymeric interactions, which limit the motion of dispersed particles and promote the transition from liquid to gel phase [17]. The possibility of direct use of plant materials would allow for a reduction of losses resulting from excessive production of some fruit and vegetables [18,19], as well as offering consumers new products based on traditionally grown raw materials [20]. Moreover, apart from fruits, such as papaya [21], different kinds of coproduct or byproducts can be used in the production of biopolymeric films, for instance from yellow passion fruit [22]. Different pumpkin species and numerous varieties differ from each other in chemical composition, color, or shape. However, usually, all parts of the pumpkin plant, including fruits, flowers, leaves, roots, stems, and seeds, are edible [23]. Pumpkins are commonly grown in many countries and are consumed as a vegetable, but can also be incorporated into many food products, such as candies, bread, and rice cake. Pumpkin contains nutrients, such as a high content of protein, peptides, dietary fiber, vitamins, and minerals [24]. Therefore, this study aimed to use pumpkin purée as a new component of plant-based materials, which provide some nutritional values for the production of packaging films, and determines the physicochemical properties of the obtained materials. Two hydrocolloids were used: highly methylated apple pectin (polysaccharide structure-forming component of plant origin) and gelatin (protein structure-forming component of animal origin). Sorption, tensile, and thermal properties of vegetable films were investigated. The chemical characteristics of pumpkin purée and the rheology of film-forming solutions were also determined.

## 2. Results

### 2.1. Selected Properties of Composite Films Determined by the Composition and Properties of Film-Forming Solutions Based on Pumpkin Purée and Selected Hydrocolloids

It is well known that the chemical composition and content of individual ingredients, or their appropriate supplementation [25,26,27,28], in film-forming solutions have a significant impact on the structural properties of the obtained film [4,15,19,29]. In the case of the studied composite film-forming solutions, the compounds ensuring the formation of the polymer matrix of the films depend not only on the hydrocolloid used, but are also significantly determined by the ingredients of the pumpkin purée in the present study. First of all, it is about compounds such as cellulose, hemicelluloses, pectins, and starch. It is also worth paying attention to lignins, which, in addition to the ability to form a polymer network, are considered to be more stable than cellulose, and their derivatives are hydrophobic. Proteins (gelatin) also contribute to the formation of the polymer matrix.

**Figure 1:**
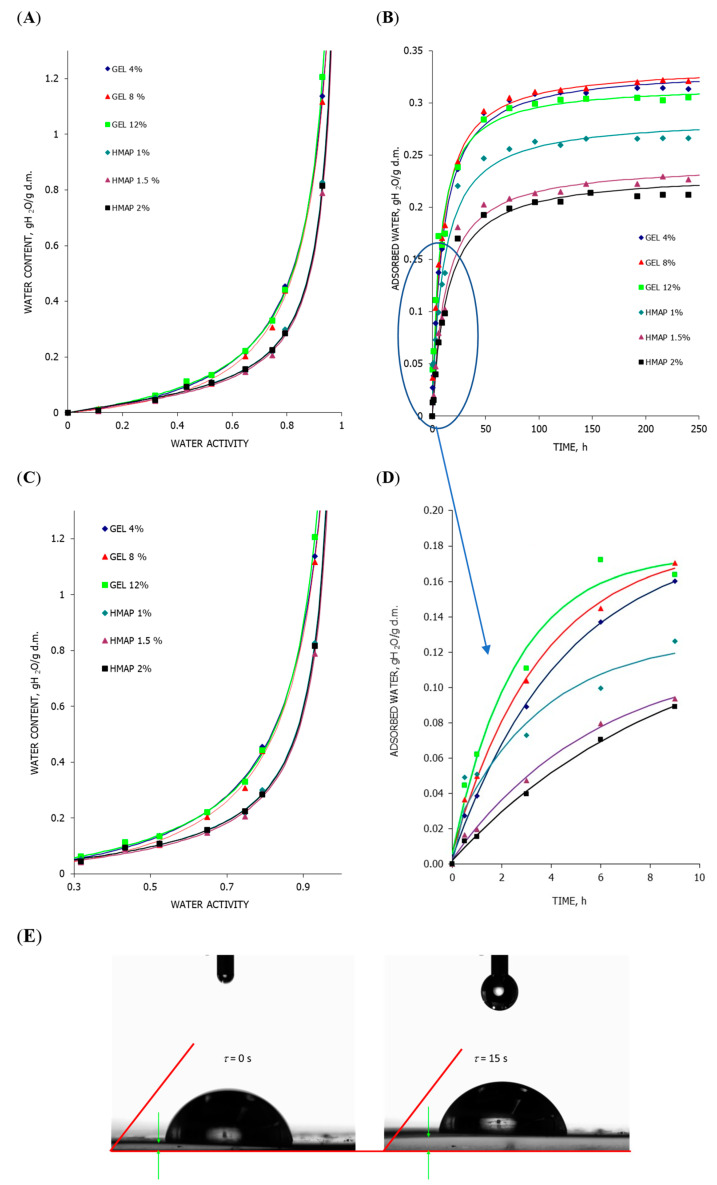
Adsorption course and kinetics of moisture absorption on the surface of composite polymers in the form of films: (**A**,**C**)—water vapor adsorption isotherm; (**B**,**D**)—kinetics of the water vapor adsorption process; (**E**)—change in the surface characteristics of protein films during the evaluation of the contact angle.

Mono- and disaccharides, in particular glucose, fructose, and sucrose, but also water, are responsible for the plasticizing effect of pumpkin purée. In addition, the mechanical properties largely depend on the size of pumpkin purée particles (Figure 1) and the content of dietary fiber (Table 1), which also significantly affects the thermal stability of the obtained films. Interestingly, however, despite the passage of years since the publication of the first reports on edible packaging based on purée of various fruits and vegetables [4,16], there is not much information on the analysis of the chemical composition of purée introduced into the polymer matrix and its impact on the physical and chemical properties of the films, and thus the final edible packaging. The authors of individual studies, analyzing the results obtained, indicate the chemical composition of the ingredients and fruit and/or vegetable purées as one of the reasons for the observed phenomena, while pointing out the need to conduct additional research in this area [5,25,26,27].

### 2.2. Physicochemical Characteristics of Composite Film-Forming Solutions

**Figure 2:**
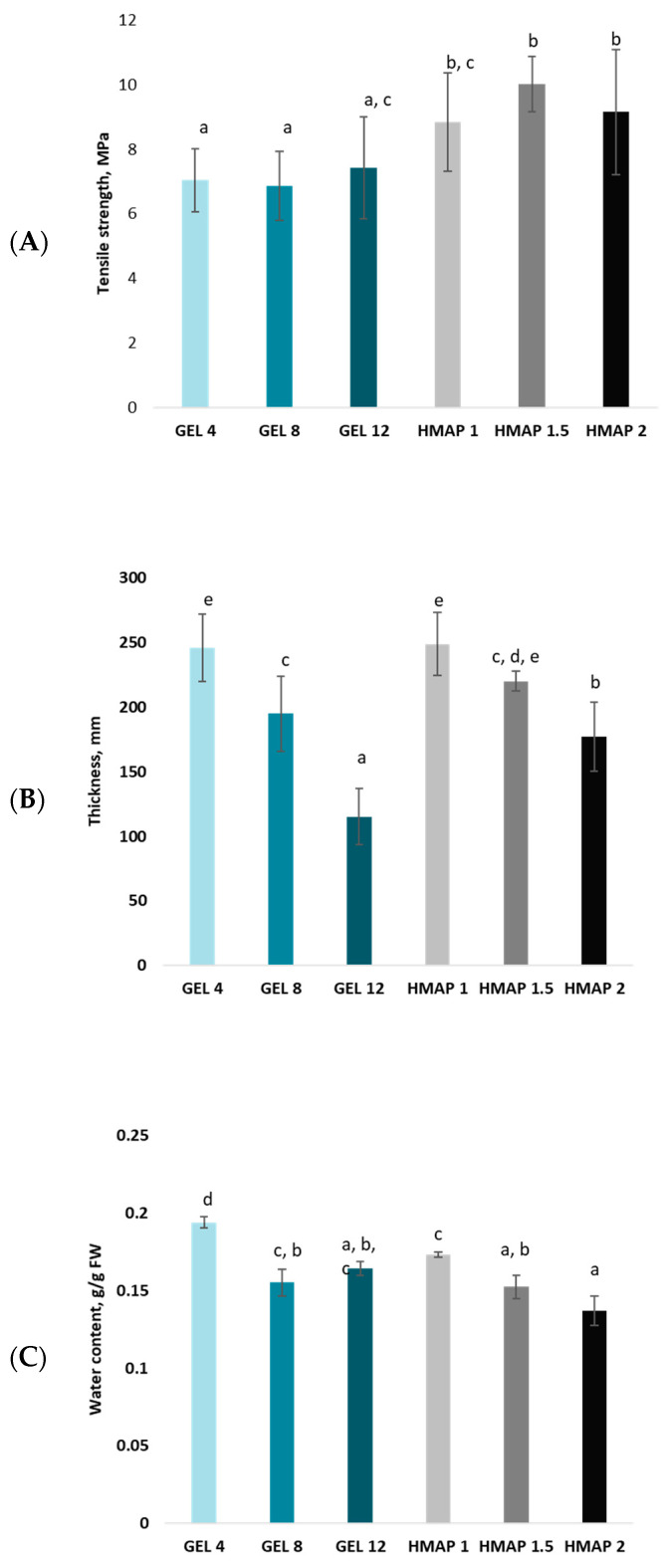
Mechanical properties of analyzed films. (**A**)—Tensile strength of tested composite films based on selected hydrocolloids with the addition of pumpkin purée. The same letters next to the values (a–c) mean no statistically significant differences (*p* < 0.05). (**B**)—Thickness of the tested composite films based on selected hydrocolloids with the addition of pumpkin purée. The same letters next to the values (a–e) mean no statistically significant differences (*p* < 0.05). (**C**)—Water content in the tested composite films based on selected hydrocolloids with the addition of pumpkin purée. The same letters next to the values (a–d) mean no statistically significant differences (*p* < 0.05).

The steam heating technique used to obtain pumpkin purée allowed a structure-forming component of film-forming solutions with specific physical properties and chemical composition, presented in Table 1. The tests and the analysis of the results allowed us to conclude that the obtained particle sizes (the smaller, the better) of the purée are suitable for obtaining composite films with the appropriate structure and functional properties. Granulometric analysis showed a bimodal particle size distribution, with two peaks near 25 µm and close to 100 µm in the volume distribution (Figure 1). The diameter D_4_._3_, which is very sensitive to the presence of large particles, was only about 80 µm. This is a satisfactory result, considering its impact on the mechanical properties of composite edible films (Figure 2A). Moreover, the purée was bright orange (Table 1), which is crucial for its appeal to future consumers. Taking into account the possibility of creating a polymer matrix, the content of water-soluble pectins was also determined, which was about 0.2 g/100 g fresh mass, starch at the level of 5.5 g/100 g fresh mass, and protein at the level of about 1.4 g/100 g fresh mass. The number of biopolymers with film-forming properties, such as pectins, starch, cellulose, hemicellulose, lignins, and proteins, was comparable to the values obtained by other authors. The analysis of the results obtained by other authors for pumpkin Cucurbita maxima [23,24,30,31], and the results obtained by our team, show that the Ambar variety is the most valuable source of film-forming biopolymers compared to other varieties. This increases the likelihood of forming self-contained edible films with satisfactory mechanical properties. At the same time, this variety is a rich source of carotenoids, which attract consumers not only with the attractive color of the product, but also with the perception of these nutrients as essential for maintaining health. In addition, pumpkin purée of the Ambar variety is a natural source of plasticizers such as glucose, fructose, and sucrose (presented in Table 1 and Figure 2A, which will be discussed in detail and confirmed by the results in Section 2.5.). It was concluded that further research is needed to determine if the amount is high enough to avoid the addition of artificial plasticizers, such as glycerol, during the production of the films. The content of L-ascorbic acid was also determined at the level of about 4.5 mg/100 g fresh mass, and carotenoids at the level of about 16 mg/100 g fresh mass, of which β-carotene accounted for about one-third. The presence of mono- and disaccharides in pumpkin purée has a significant impact on the elasticity of the obtained edible films. The content of total sugars was determined at the level of about 3.7%, and the content of reducing sugars at the level of about 2.2%. Glucose, fructose, and sucrose, the content of which ranged from about 1 to 1.4 g/100 g fresh mass, were responsible for the plasticizing effect of the purée (Figure 2A). Taking into account the content of ingredients determined both by the authors of this study and other studies conducted around the world, pumpkin seems to be a source of many valuable chemical compounds, including vitamins, minerals, unsaturated fatty acids, and carotenoids. Its health benefits have been known for thousands of years when it was used in traditional medicine to treat many diseases. Current research results confirm the broad spectrum of pumpkin’s health-promoting effects. It is indicated primarily for hypolipemic and hypoglycemic effects. As scientific research shows, the health-promoting properties of pumpkin result from the presence of many bioactive compounds. Carotenoids are among them. The most numerous fraction are beta- and alpha-carotene. In addition, pumpkin has antimicrobial properties. Dishes prepared based on pumpkin, and products enriched with it, can be a valuable element of the usual human diet [3,30,31,32].

### 2.3. Analysis of the State of Water in the Polymer Matrix of Composite Gelatin and Pectin Films with the Addition of Pumpkin Purée

Water vapor adsorption capacity is a characteristic feature of a given product, and depends on its structure and chemical composition. Hygroscopic properties are related to the ability to absorb water in a humid environment or release water in a dry environment. As a consequence, this affects the change in water content in the product, which determines the durability of food. The rate of adsorption and desorption depends on the affinity between the diffusing molecule and the components of the film, especially in the case of water migration. The water vapor adsorption isotherm is a relationship used in the study of the adsorption equilibrium. It determines the equilibrium relationship between the amount of water adsorbed by a unit of mass of a food product and the activity of water at constant temperature and constant total pressure. Figure 1 shows the course of water vapor adsorption isotherms (A) and the kinetics of changes in water content over time (B) for composite gelatin and pectin films with the addition of pumpkin purée. The nature of changes in water content as a function of its activity, regardless of the type of hydrocolloid and its concentration, indicates that composite gelatin films with the addition of pumpkin purée are characterized by a greater ability to adsorb moisture from the surrounding environment (Figure 1A), which is also confirmed by the curves showing the course of changes in water content in time (Figure 1B). At the same time, the kinetics of water content changes in the tested composite materials (Figure 1B) indicate the influence of the type and concentration of hydrocolloid on the course of these changes, particularly visible in the case of pectin films. Moreover, it was observed that the nature of the changes in water vapor adsorption in the case of films, in which different concentrations of animal-derived hydrocolloid (gelatin-PGEL) were used, is different in the first hours compared to films based on plant-derived hydrocolloid (pectin-HMAP), and changes significantly after 10 h of the films staying in an environment with a relative humidity of RH = 75.3%. It was observed that in the first 10 h, the amount of adsorbed water vapor is higher for gelatin films with a higher hydrocolloid content (Figure 1D), which is also observed for pectin films. Then, probably due to the rapid filling of the active centers with water molecules adsorbed on the film surface, and by the swelling of protein structures embedded in the polymer matrix, which can hold larger amounts of water, not only does the film surface swell, but also slows down the absorption of water vapor and its bonding on the surface of the material with a higher concentration of gelatin, which confirms the course of the water vapor adsorption isotherm (Figure 1A,C). The described phenomenon seems to be confirmed by the unpublished research by the authors of this article on composite gelatin films of various origins obtained using animal broth with an average protein content of 14–16%, measuring the contact angle as a function of time on the surface of composite protein polymers (Figure 1E). During the measurement (*τ* = 0 and *τ* = 15 s), no change in the contact angle was observed, which should be proof of the barrier properties of the tested films, but swelling of the polymer matrix surface blocking the possibility of moisture diffusion into the structure was observed. Coatings may lose their good mechanical and barrier properties when in contact with steam or water, and potentially swell [33]. Swelling is an important parameter for polysaccharide films because it indicates a correlation between the amount of adsorbed moisture from the environment and the rate of this phenomenon on the surface of the investigated polymer matrix, which could be characterized by a more homogeneous structure and the presence of layered crystal structures formed as a result of the interaction of pumpkin purée and highly methylated apple pectin. However, in this type of crystalline network building, the matrix structures are not able to retain too much moisture [34,35], hence the composite pectin films were characterized by moisture adsorption curves in the range of lower water content compared to gelatin films [36]. At the same time, in the case of hydrophilic films, the permeability to gases increases with the increase in the degree of hydration, which is caused by the plasticization of the polymer network, which facilitates diffusivity and water penetration, and may limit the practical use of this type of polymer [11]. When creating edible films and coatings that are moisture barriers, it is often necessary to use materials that are completely insoluble in water, to avoid loss of barrier properties of the films due to contact with high water activity foods. In such cases, it is worth including lipids or proteins in polysaccharide films, which in the case of composite gelatin films with the addition of pumpkin purée has not been confirmed in the conducted research and the results discussed. It was noted in the research of Galus and Kadzińska [37] that the moisture content at higher values of water activity (aw > 0.6) begins to increase exponentially, causing structural changes in the film that enable the phenomenon of facilitated water transport. The presence of rapeseed oil leads to changes in the structure of protein films, which affect the affinity of these films for water, changing their properties. At constant aw, the equilibrium moisture content in the analyzed protein films remained constant and did not change with increasing temperature. A different internal arrangement of the structure in the cross-section of the films as a function of their composition was observed. Experiments have shown that changing the composition of protein films will not significantly affect the appearance of food products during use. The addition of rapeseed oil could reduce sensitivity to moisture, which would be particularly important when using the film to protect products with high humidity. On the other hand, studies of composite polymer gelatin films with the addition of purée allowed to obtain the final water content in the material at the level of 0.313 ± 0.008 gH_2_O/g d.m., which was lower than that obtained by Galus and Kadzińska [37]. However, the course of the isotherm indicated the same mechanism of the binding process of water on the active surface of the tested composite gelatin films with the addition of pumpkin purée. On this basis, it can be assumed that the inclusion of pumpkin purée in the protein matrix introduced components limiting the amount of adsorbed moisture into the polymer matrix, and could improve the performance properties of the tested gelatin composites. Protein films are hydrophilic in nature. The presence of hydrophilic groups in the spatial structure of proteins affects the significant water vapor permeability of protein materials. In an environment with high relative humidity, water sorption causes cross-linked polymers to swell, which facilitates the migration of water through the film, and thus significantly reduces its barrier properties [38]. Protein films are characterized by a very good barrier to polar compounds. However, their hydrophilic nature affects the high solubility of this type of material in water and their swelling, which is confirmed in Figure 1E. Research is being conducted to improve the physical properties of protein films, and one of the methods used for this purpose is the addition of various types of plant components and more [4,39], as in this work. The interpretation of water vapor adsorption kinetics is possible due to the dependence on the water content increase in the tested material at the time of the sorption process. The shape and location of the obtained curve depend on factors such as the type of material tested and the measurement parameters (relative humidity of the environment and temperature). Galus and Lenart [38] studied the effect of glycerol on the kinetics of water vapor adsorption by whey coatings. The water adsorption process was the fastest in the initial phase of the study, i.e., for the first 5 h. After this time, a significant slowdown in this process was observed. The hydrophobicity and hydrophilicity of the individual components of the composite polymer structures affect the affinity of edible films and coatings to moisture or directly to water in terms of their use as edible packaging with a protective function. The theory is also confirmed for films based on polysaccharides, but the trend is not so evident.

### 2.4. Analysis of the Mechanical Parameters of the Polymer Matrix of Composite Gelatin–Pectin Films with the Addition of Pumpkin Purée

Standardized requirements for mechanical properties are dedicated to polymer structures in the context of their protective function. Tensile strength and elongation at break are the most important parameters that are crucial for studying the mechanical properties of edible films and coatings. Such tests may also include modulus of elasticity, compressive and flexural strength, stiffness, bursting strength, and abrasion resistance. These properties are very important for the film-forming material because they determine its strength, flexibility, and protective capabilities. Good mechanical properties of films are among the basic requirements that must be met in food packaging, because poor flexibility or strength can lead to premature failure or cracking during production, handling, storage, or use [4,18]. The mechanical properties of the films can be improved through the use of appropriate pretreatment of film-forming solutions, or the appropriate selection of components for the production of composite polymer structures through the appropriate synergistic action of ingredients that may affect the functional and operational properties of the films or coatings produced. The addition of components containing polysaccharides to the protein film/coating can improve the moisture resistance and mechanical properties of the polymer. The strong interaction between the ingredients results in the formation of an ordered structure and a protein–polysaccharide network, which leads to improved mechanical and barrier strength [29].

All of the tested composite films based on selected hydrocolloids with the addition of pumpkin purée were characterized by good mechanical strength, and the obtained parameters ranged from about 7 to over 10 MPa (Figure 2A). The statistical analysis showed statistically significant differences in the tensile strength values in terms of the use of hydrocolloids, without confirming the significance of the effect of their concentrations, except for the 1% concentration of pectin (HMAP 1) and the about 12% concentration of gelatin (GEL 12). However, differences in mechanical properties could also be influenced by differences in the water content in the obtained composite polymers (Figure 2C). The analysis of the statistical results allowed us to conclude that there is a positive correlation between the film thickness and the water content in terms of the impact on the composite strength, only in the case of the material obtained by structuring with a 4% addition of gelatin. In the remaining cases, the trends observed are difficult to explain, but it is worth noting that the composite polymer in the form of a gelatin film with the smallest thickness (GEL 12—Figure 2B), characterized by water content not significantly different from the others, was characterized by strength significantly similar to pectin films, whose strength was statistically significantly different from gelatin films. At the same time, it was noted that in the case of films with the lowest concentration of hydrocolloid, the thickness of the structure corresponds to the higher water content in the polymer matrix in the case of polysaccharide films, and is inversely proportional to the hydrocolloid concentration. Unfortunately, in the case of composite gelatin films, the correlation between thickness and water content was confirmed only in the case of a hydrocolloid concentration of 4%. According to the standards, for edible coatings to be used as biodegradable packaging materials, they should have a tensile strength above 3.5 MPa [40]. In connection with the above, regardless of the correlations and interactions between the components within the composite structures produced in the polymer matrix, all of the tested films meet the condition related to tensile strength, and may be considered biodegradable packaging materials.

### 2.5. Analysis of the Thermal Properties of the Polymer Matrix of Composite Gelatin–Pectin Films with the Addition of Pumpkin Purée

The thermal characteristics of edible films from purée solutions of gelatin and highly methylated apple pectin, and gelatin and pectin with the addition of pumpkin purée, are summarized in Table 2.

**Figure 3:**
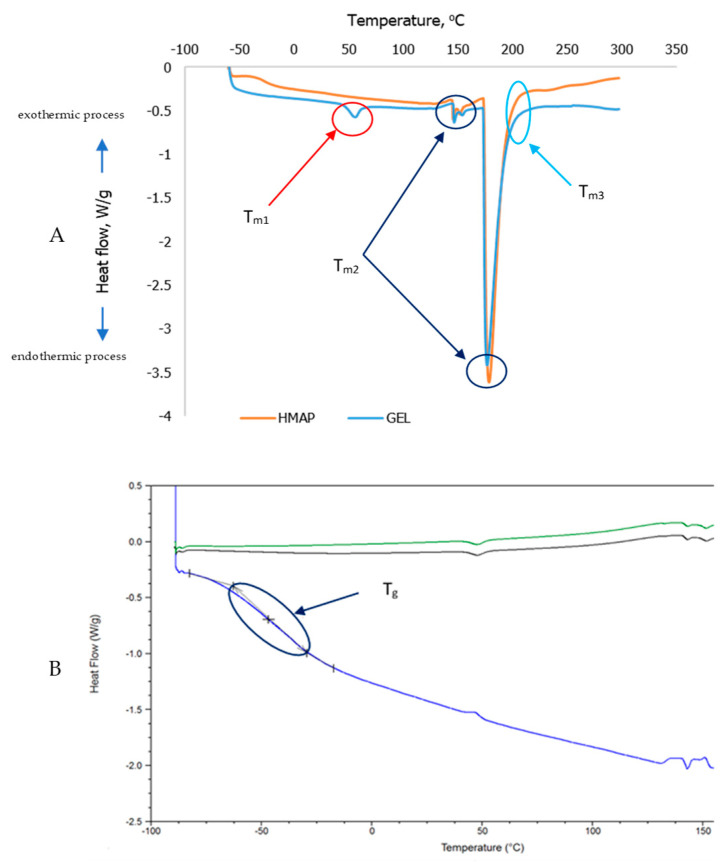
Examples of thermograms for tested composite films based on selected hydrocolloids with the addition of pumpkin purée: (**A**)—DSC; (**B**)—MDSC.

Changes in the glass transition temperature (Tg), melting point (Tm), and enthalpy (ΔH) are the best indicators of the compatibility of different biopolymers. Thermography showed that composite films based on gelatin, highly methylated pectin, and pumpkin purée are subject to a multistage degradation process. In general, protein films, compared to polysaccharide films, were characterized by lower melting point values. In the case of gelatin films, in all samples with different concentrations of gelatin, we observed that the first endothermic transition occurred at about 60 °C and was responsible for the gelatin melting process (Table 2, Figure 3A).

The next two peaks indicate the process of polymorphic transformations of sugars such as glucose, fructose, sucrose, starch, and pectins. It can be assumed that the lower values of gelatin melting point when the concentration of this protein in polymers in the form of films was at the level of 8 and 12%, was because it was less protected by saccharides due to their lower content in the composition of the finished composite film. For all composite polysaccharide films with highly methoxylated apple pectin, regardless of its concentration, endothermic peaks were also observed, but they were responsible for the polymorphic transformations of saccharides and occurred in the temperature range from about 145 to slightly above 156 °C (Table 2, Figure 3A). As the percentage of pectin rises, the temperature range of the endothermic peaks also shifts towards higher temperatures. However, the shifts in saccharide transformations are statistically insignificant. The statistical analysis confirmed that there were no significant differences between the various types of hydrocolloids studied. However, it was observed that there was a slight increase in the melting point value of composite pectin polymers when the concentration of hydrocolloid was increased, specifically in those with pumpkin purée added.

On the DSC curves of edible films made with pumpkin purée, the first endothermic peak was responsible for the phenomenon of gelatin melting and ranged from over 57 to about 67 °C, depending on the hydrocolloid concentration in the tested composite polymers with the addition of pumpkin purée (Table 2, Figure 3A). The DSC curves of composite films with pumpkin purée did not show any exothermic peaks. This suggests that the vegetable component’s biopolymers may have a protective effect on the sample proving its high compatibility with the hydrocolloids used, creating well-structured films. Similar observations were made by Orsuwan et al. [10] for edible agar foils made from banana powder. The authors indicated that all blended films showed only one peak without phase separation. The two endothermic peaks observed at a temperature above 100 °C for the film with apple purée, and with and without vegetable oils, are related to the phenomenon of polymorphic transformations of various carbohydrates, including monosaccharides such as glucose and fructose, and pectins. Iijima et al. [41] observed an endothermic melting peak of pure pectin at 154 °C, while Athmaselvi et al. [42] provided DSC curves for guava powder, where they observed endothermic peaks centered at 208.66 °C, 341.16 °C, and 500.75 °C; this is associated with the melting of pectin contained in guava and the possibility of demethoxylation, dihydroxylation, and decarboxylation of pectin and other polysaccharides. The values of the melting point of the membranes obtained in this study may also be related to the melting of sugars such as glucose, fructose, and sucrose, which are present in large amounts in apple purée. The obtained melting point values are consistent with those for crystalline sugars of analytical purity, which were studied by Saavedra-Leos et al. [43], who also reported values of degradation temperatures between 195 and 235 °C for fructose and sucrose, respectively. The authors of the study did not observe peaks at these temperatures and suspected a protective effect of the natural mixture of various carbohydrates and proteins present in apple purée on the thermal stability of edible films. The Tg temperature of food materials ranges from −135 °C for water to about 200 °C for some anhydrous biopolymers [44,45]. MDSC analysis found glass transition temperature (Tg) values that were very low (Figure 3B), ranging from about −34.6 to about −46.5 °C, suggesting that these materials are not in a glassy state at room temperature (~25 °C), the temperature used in the second drying step, in the conditioning, as well as in all characterizations (Table 2, Figure 3B). According to Chang et al. [46], plasticization weakens the intermolecular forces between the polymer chains and consequently reduces the overall cohesion, reducing the Tg. Lower values of the glass transition temperature are related to the presence of low molecular weight natural plasticizers in the apple purée, such as fructose, glucose, and sucrose, as well as to the plasticizing effect of vegetable oils. Such low Tg values were also caused by the presence of water (values of water activity of the examined layers oscillated around 0.5). The phenomenon with only one Tg observed in mixed polymers in the DSC scan indicated good compatibility of the component biopolymers with pure sodium alginate chemically isolated from raw materials [7].

To test composite films with pumpkin puree, we utilized the DSC and MDSC method to determine thermal parameters such as melting point, polymorphic changes, glass transition temperature, and decomposition temperature. These parameters were analyzed based on various factors, including their use as packaging materials. When examining composite gelatin or pectin films with pumpkin puree, we found that the combination of hydrocolloid and vegetable components had a synergistic effect on the structure’s properties.

### 2.6. Analysis of the Structure of the Polymer Matrix of Composite Gelatin–Pectin Films with the Addition of Pumpkin Purée

**Figure 4:**
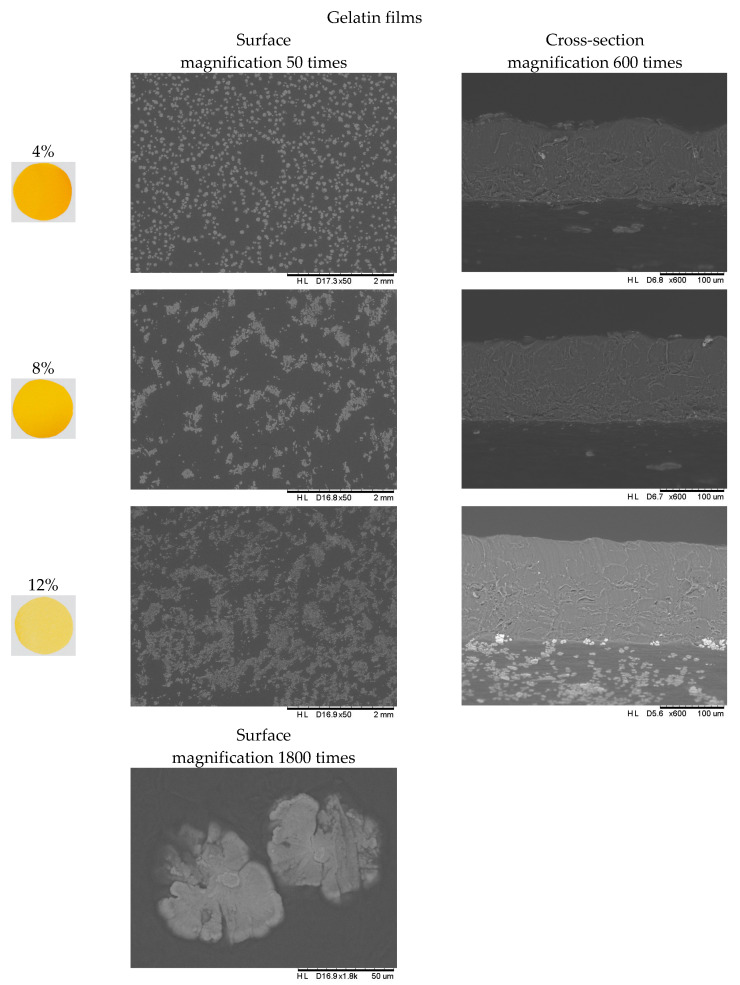

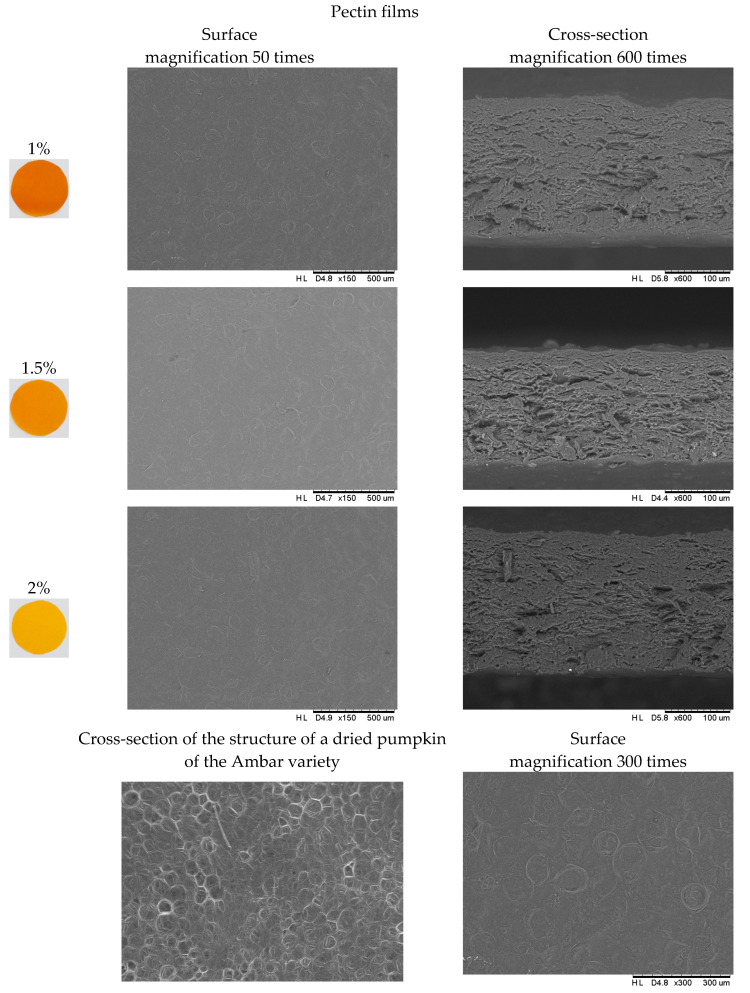
Structure of the polymer matrix of composite gelatin and pectin films with the addition of pumpkin purée.

The structure is an important feature of food, and is the basic factor influencing the assessment of the quality of raw materials and food products. It plays a key role in the consumer’s perception of food when choosing a finished product, which is why food manufacturers try to preserve its appropriate form. Microscopes are used more and more often to study the physical properties of food due to noninvasive techniques for imaging structure features. Taking digital photographs allows for the assessment of the tested surface of the material, which may also allow for the assessment of the quality of films and edible coatings in terms of their properties as edible packaging. Figure 4 shows the surface structure and the cross-sectional area of the tested composite polymers in the form of films with the addition of pumpkin purée. Based on the visual analysis of documentation from the electron microscope, it was found that clusters of pumpkin purée particles are present on the surface of the gelatin films, the accumulation of which on the surface of the composite matrix clearly distinguishes it from the surface of composite matrix films based on pectin with the addition of pumpkin purée. At the same time, the presence of purée particles on the surface is not visible to the “naked eye” on the surface of the resulting composite polymer in the form of a film. However, the films brighten with increasing concentrations of hydrocolloids, regardless of their type. The figure also shows a photo of the structure of the dried pumpkin and, after examining the surface structure of the pectin-based films with the addition of purée, it can be concluded that the structure of the pectin films resembles the surface of the dried vegetable, on which one can observe the distribution of spaces identified as cells limited by the cell wall fixed as a result of drying. To better visualize the trends, the picture shows the surface of the pectin film with the addition of pumpkin purée, magnified 300 times. Based on the observations made during the structure analysis, it can be concluded that the cross-sectional structure of gelatin films is characterized by a more uniform and smooth surface compared to pectin films, in which significant cracks and irregularities, as well as empty spaces in the matrix of composite pectin polymers, are observed. The result of the formation of this type of heterogeneous structure of pectin films is their functional properties, such as material strength (Figure 2A) and limited diffusivity, in this case, tested based on moisture adsorption in variable conditions of water activity (Figure 1). This is also associated with the presence of less incorporation into the polymer matrix of amorphous domains formed during the drying and conditioning of films with the addition of pumpkin purée, which is largely responsible for the presence of these domains in the structure of the studied polymer materials.

## 3. Materials and Methods

### 3.1. Materials

The research material consisted of film-forming solutions and films from pumpkin fruit purée (*Cucurbita maxima*) with the addition of highly methylated apple pectin (HMAP) (Naturex, Warsaw, Poland) or pork gelatin with BLOOM 180 (PGEL) (Agnex, Białystok, Poland). Pumpkins of the Ambar variety were harvested at the experimental field ‘Wolica’ of Warsaw University of Life Sciences—SGGW, and were stored at a constant temperature of 16 °C before use. Anhydrous glycerol (purity 99.5%), calcium chloride, and salts used to prepare saturated solutions for the determination of water vapor sorption isotherms (lithium chloride, potassium acetate, magnesium chloride, potassium carbonate, magnesium nitrate, sodium nitrate, sodium chloride, sulfate ammonium, barium chloride) and other chemicals necessary for the chemical analysis were supplied by Avantor Performance Materials Poland S.A. (Gliwice, Poland).

### 3.2. Characteristics of Pumpkin Purée

#### 3.2.1. Water Content

The water content was determined based on the determination of the dry matter content. The dry matter content was determined by the gravimetric method. On an analytical balance, AE240 (Mettler-Toledo International, Inc., Greifensee, Switzerland), 2 g of film-forming solution and/or pumpkin purée was weighed in weighing bottles with an accuracy of 0.0001 g. The samples prepared in this way were dried with sand in a laboratory dryer with an electronic temperature regulator, type SUP-65 W/G (WAMED, Warsaw, Poland), at 70 °C for 24 h. The vials were cooled in a desiccator with CaCl_2_ and weighed again. The assay was performed in triplicate. The water content was calculated from the formula:

$$u=\frac{m_{1}-m_{2}}{m_{2}}$$

where *u*—water content, (g H_2_O/g_d_._m_.); *m*_1_—mass of the sample before drying, (g); *m*_2_—mass of the sample after drying, (g).

#### 3.2.2. Total and Active Acidity

Total acidity was determined by the potentiometric method in triplicate. The active acidity (*pH*) was determined using a LAB 850 *pH* Meter (SHOTT, Mainz, Germany). The assay was performed in three replicates.

#### 3.2.3. Extract

The extracted content in pumpkin purée was determined using an electronic refractometer and expressed in Brix. The determination was performed in three repetitions.

#### 3.2.4. Chemical Composition

The content of total sugars, reducing sugars, glucose, fructose, sucrose, lactose, and maltose (the so-called sugar profile) was determined using the high-performance liquid chromatography method with refractometric detection (HPLC-RID). Starch content was determined using the Luff–Schoorl titration method. The protein content was calculated by determining the nitrogen content by the Kjeldahl method. The content of water-soluble pectins was determined according to the International Federation of Fruit Juice Producers method no. 26. Fiber, lignin, cellulose, and hemicellulose contents were determined by the gravimetric method using a Fibertec System M 1017 Hot Extractor and a Fibertec Systems M 1018 Cold Extractor (Foss, Hilleroed, Denmark). Total carotenoids, β-carotene, and vitamin C were determined using the high-performance liquid chromatography method with UV–VIS detector method. All measurements were performed in three replicates.

#### 3.2.5. Color

The color of the pumpkin purée was determined in the CIE L*a*b* system using a Konica-Minolta CM-5 colorimeter (Konica Minolta, Japan) with diffuse illumination (measuring geometry d/8°, CIE 2° standard observer, D65 illuminator). The measurement was performed in ten repetitions. Total color difference (Δ*E*) was calculated from the following equation [6]:

$$\Delta E=\sqrt{{\left(L^{*}-L\right)}^{2}+{\left(a^{*}-a\right)}^{2}+{\left(b^{*}-b\right)}^{2}}$$

where Δ*E*—total color difference, (−); L*, a*, b*—measurements of brightness and trichromatic components for fresh pumpkin, (−); *L*, *a*, *b*—measurements of brightness and trichromatic components for the sample, (−).

### 3.3. Preparation of Film-Forming Solutions

Aqueous film-forming solutions were prepared from highly methylated apple pectin at the concentration of 1, 1.5, and 2%, or pork gelatin at the concentration of 4, 8, and 12%. The solutions were heated for 30 min at 80 °C, then cooled down to 50 °C, and glycerol was added in the amount of 50% of the weight of the hydrocolloid. Pumpkin purée was prepared by cooking (10 min with 10% of water) and homogenizing, and then it was added to solutions in a constant amount of 40% and homogenized. In the next stage, the film-forming solutions were degassed in a vacuum dryer for 1.5 to 2 h at a pressure of 130–230 hPa depending on the film-forming composition. The additions of protein hydrocolloid in the form of gelatin at the level of 4, 8, and 12% (*w*/*v*), and polysaccharide hydrocolloid at the level of 1, 1.5, and 2% (*w*/*v*), were selected based on data in the literature [20,25].

### 3.4. Particle Size Distribution in Film-Forming Solutions

Particle size analysis of the film-forming solutions as a granulometric distribution of solid particles in water was performed in three repetitions using a Mastersizer 2000 particle size analyzer (Malvern Instruments, Worcestershire, UK). Characteristic parameters of the distribution, such as *DV*_10_, *DV*_50_, and *DV*_90_ diameters corresponding to 10%, 50,% and 90%, respectively, of the volume (mass) of the set of particles were determined. The equivalent diameters *D*_3.2_ and *D*_4.3_ were calculated according to the equations:

$$D_{3.2}=\frac{\sum d^{3}}{\sum d^{2}}$$

$$D_{4.3}=\frac{\sum d^{4}}{\sum d^{3}}$$

where *d*—diameter of a single solid particle, (µm).

Equivalent diameter *D*_3.2_ defines the diameter of a sphere equivalent in area, whereas equivalent diameter *D*_4.3_ defines the diameter of a sphere equivalent in volume or mass.

### 3.5. Film Formation

The deaerated film-forming solutions were poured onto Teflon sheets so that each 1 square centimeter of substrate contained 0.03 g_d_._m_., which made it possible to compare films with different concentrations of added hydrocolloids. The spilled solutions were then dried at 120 °C for 1.5 h to reduce the overall drying time, and also to avoid possible spoilage of the solutions. After initial drying, the spilled film-forming solutions were transferred to the climatic chamber, where additional drying was carried out at 25 °C for 72 h and relative humidity (RH) of 50 ± 1%. After this time, the films were removed and stored in the same conditions (T = 25 °C, RH = 50 ± 1%) for 7 days before performing the analyses.

### 3.6. Water Vapor Sorption Isotherms and Kinetics

Water vapor adsorption isotherms of edible films were determined using the static method. The tests were carried out in the range of water activity from 0.000 to 0.934 at 25 °C. The samples were stored in desiccators for 15 days until they reached equilibrium with the surrounding atmosphere (preliminary tests showed that the samples reached a constant weight after about 10 days). The assay was performed in three repetitions for each type of film. The equilibrium water content in hydrocolloid–pumpkin edible films after the adsorption process was calculated from the formula [47]:

$$u=\left(\frac{d}{c\times \frac{b}{a}}-1\right)\times 100$$

where *u*—equilibrium water content, (g H_2_O/100 g_d_._m_.); *a*—initial weight of the sample from the desiccator with CaCl_2_, (g); *b*—a final mass of the sample from the CaCl_2_ desiccator, after 15 days in the CaCl_2_ desiccator and after drying in a vacuum oven at 70 °C and 1.1 kPa pressure for 24 h, (g); *c*—the initial weight of the sample from the desiccator with the specified solution, (g); *d*—final weight of the sample after 15 days of storage in a desiccator with a specific solution, (g).

### 3.7. Mechanical Properties

Mechanical properties testing was performed using a TA XT2i texture meter (Stable Micro Systems Ltd., Surrey, UK). The tensile strength test of the coatings was tested according to ASTM D882–02 [48]. A minimum of 10 repetitions were performed for each type of coating. The tensile strength (*TS*) elongation at break (*E*) was calculated according to the equations:

$$TS=\frac{F_{max}}{A}$$

where *F_max_*—force causing shell rupture [N], *A*—cross-sectional area of the shell before the tensile test [mm^2^], and

$$E=\frac{\Delta l}{l_{0}}\times 100$$

where Δ*l*—elongation of the sample at which the film was broken [mm], *l*_0_—the initial length of the sample before the tensile test [mm].

The experiment involved placing 2.5 × 10 cm samples between two measuring jaws that moved apart at a constant speed of 1 mm/s to a distance of 25 mm until the test sample broke off. The system recorded parameters such as load and elongation of the foil, which were then used to create curves defining the maximum force that caused the foil to break, and its elongation. We determined the tensile strength of the tested material by using the maximum force per unit of the cross-sectional area of the foil subjected to measurements. The elongation of the foil was calculated by measuring the elongation of the measuring section and the distance between the jaws used at the beginning of the measurement.

### 3.8. Thermal Properties

Differential scanning calorimetry (DSC) and modulated differential scanning calorimetry (MDSC) using TA Instrument Q200 differential scanning calorimeter (TA Instruments, New Castle, DE, USA) were determined to obtain heat flow (W/g) versus temperature curves and the glass transition temperature for analyzed vegetable films, respectively. The diagrams were analyzed concerning the total, reversible, and nonreversible heat flow. Glass transition (Tg) was determined as the midpoint of a vertical shift in the reversing transition curve. All analyses were completed in triplicate.

### 3.9. Microstructure

The surface morphology of the films was observed using the scanning electron microscope TM-3000 HITACHI (Hitachi High-Technologies Corporation, Chiyoda, Tokyo, Japan). Samples were coated with gold under vacuum using the coater Cressington 108 Auto (Cressington, Watford, UK). A 5 mm × 5 mm film was fixed on the support using silver paste. An accelerating voltage of 15 kV and a magnification of 500× were used.

### 3.10. Statistical Analysis

Statistica 13.1 software with Spearman’s R correlation at a significance level of α = 0.05 were used to determine the correlation between samples. In addition, based on the gelation temperature of the solutions, homogeneous groups were determined using the Tuckey test (α = 0.05). The obtained results were also subjected to a two-factor analysis of variance to determine the influence of the type and concentration of hydrocolloid on the properties of the composite films obtained.

## 4. Conclusions

Research has been conducted on the potential use of pumpkin purée as an ingredient in the creation of composite packaging polymers in the form of gelatin and pectin films. The evaluation of the composition allowed for the interpretation of the results obtained at the stage of discussing the performance properties of the tested composite films. The course of the isotherm indicated the mechanism of the water binding process on the active surface of the tested composite gelatin and pectin films with the addition of pumpkin purée. It can be assumed that the incorporation of pumpkin purée into the protein matrix introduced components limiting the amount of adsorbed moisture into the polymer matrix, and could improve the functional properties of the tested gelatin composites. The analysis of the results allowed us to conclude that there is a positive correlation between the thickness of the film and the water content in terms of the impact on the strength of the composite in the case of the material structure with the addition of gelatin. Glass transition temperature and aw are independent parameters that affect food stability. A better understanding of the relationship between food microstructure and heterogeneity in the glass transition properties and water content is required to further understand the importance of the glass transition and aw in controlling food stability and edible packaging. The results have shown that pumpkin is a valuable plant material that, when used in purée form, can provide essential ingredients to enhance the composition of edible films. These films can then be used as protective packaging for food products. All of the tested composite films were characterized by good mechanical strength. It was shown that the thermogram courses vary depending on the origin of the hydrocolloid. This is due to the cross-linking of the polymer matrix structure caused by gelatin, highly methylated apple pectin, and biopolymers found in pumpkin purée.

## Figures and Tables

**Table 1:** Physical and chemical properties of pumpkin purée used as a structure-forming ingredient in film-forming solutions.

Parameter	Symbol/Unit	Value
Particle size distribution	*D*_4.3_ [μm]	80.47 ± 1.49
	*D*_3.2_ [μm]	52.69 ± 1.70
	*DV*_10_ [μm]	26.30 ± 1.63
	*DV*_50_ [μm]	80.28 ± 1.47
	*DV*_90_ [μm]	125.07 ± 3.31
Color 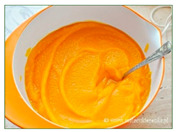	*L* [−]	54.61 ± 0.07
	*a* [−]	25.47 ± 0.04
	*b* [−]	83.40 ± 0.14
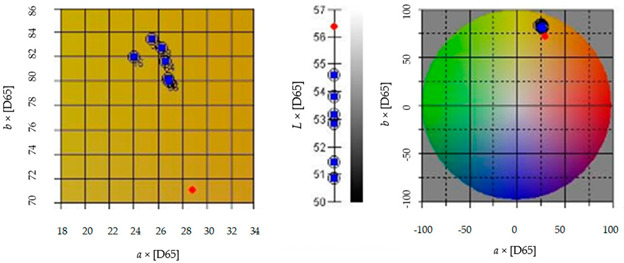		
Water content	*u* [g H_2_O/g _d_._m_] [%]	8.52 ± 0.27 89.5 ± 0.3
Total soluble solids	*E* [°Brix]	8.3 ± 0.1
*pH*	*pH* [−]	7.3 ± 0.2
Pectins soluble in water	g/100 g _fresh mass_	0.23 ± 0.01
Starch	%	5.54 ± 1.91
Cellulose	%	4.6 ± 0.3
Hemicellulose	%	8.5 ± 0.6
Lignin	%	13.1 ± 0.4
Fiber	%	1.96 ± 0.36
Protein	%	1.38 ± 0.16
L-ascorbic acid	mg/100 g _fresh mass_	4.47 ± 0.01
Total carotenoids	mg/100 g _fresh mass_	15.96 ± 0.52
Β-carotene	mg/100 g _fresh mass_	4.77 ± 0.39
Total sugars	%	3.71 ± 0.46
Reducing sugars	%	2.23 ± 0.10
Glucose	g/100 g _fresh mass_	1.38 ± 0.20
Fructose	g/100 g _fresh mass_	1.08 ± 0.10
Saccharose	g/100 g _fresh mass_	1.24 ± 0.05

**Table 2:** Parameters determined during thermogravimetric analysis for gelatin and pectin films with the addition of pumpkin purée.

Sample	T_m1_ [°C]	T_m2_ [°C]	T_m3_ [°C]	ΔH [J/g]	Tg	Total Mass Loss in the Temperature Range of 50–300 °C [%]
GEL4	66.86 ± 0.91 ^2^	150.89 ± 7.52 ^a,2^	177.85 ± 10.15	214.35 ± 20.44	−39.99 ± 0.99 **	42.38 ± 0.58 *
GEL8	57.80 ± 1.61 ^1^	146.99 ± 0.04 ^a,1^	175.76 ± 14.80	201.40 ± 13.08	−46.45 ± 0.66 ***	45.33 ± 0.08 **
GEL12	59.16 ± 5.53 ^1^	158.97 ± 6.35 ^a,2^	189.42 ± 15.72	176.13 ± 62.39	−45.58 ± 0.81 ***	41.97 ± 0.08 *
HMAP 1		145.38 ± 0.11 ^ACa^	177.02 ± 1.73	248.40 ± 0.57	−34.64 ± 0.27 *	46.04 ± 0.43 ***
		152.31 ± 0.66 ^BCa^				
HMAP 1.5		148.63 ± 1.82 ^ACa^	179.49 ± 1.27	262.75 ± 2.05	−34.81 ± 0.59 *	46.27 ± 0.29 ***
		154.17 ± 0.98 ^BCa^				
HMAP 2		149.99 ± 1.02 ^ACa^	180.34 ± 1.70	245.70 ± 1.05	−39.41 ± 0.08 **	47.25 ± 0.12 ****
		156.05 ± 0.96 ^BCa^				

^A,B^ Statistical significance of the influence of HMAP concentration, ^C^ significance of temperature variability in the course of the curve, ^1,2^ statistical significance of the influence of GEL concentration and ^a^ significance the influence of the type of hydrocolloid; * determination of group homogeneity (the number of characters differentiates homogeneous groups)—influence of hydrocolloid type and concentration (for total mass loss).

## Data Availability

Samples of the compounds and all data are available from the authors.

## Sample Availability

Samples of the compounds are available from the authors.
